# Toward a US Army Pacific (USARPAC) rapid deployment medical component in support of Human Assistance/Disaster Relief (HA/DR) operations: challenges with “Going in Light”

**DOI:** 10.1186/s40696-016-0025-4

**Published:** 2016-10-26

**Authors:** Ralph J. Johnson

**Affiliations:** 1 United States Army, Medical Operations and Planning Office, HQ, USARPAC, G-3 HADR, BLDG x348, Fort Shafter, HI 96858 USA; 2 1st BDE, 1 Southern Div, 75th Trng Cmd, 10949 Aerospace Ave., Houston, TX 77034 USA

**Keywords:** Military disaster preparedness, U.S. Army Pacific, Emergency medical disaster response

## Abstract

**Background:**

This article reports the exploratory development and study efforts regarding the viability of a novel “going-in light” or “Going Light” medical component in support of US Army Pacific (USARPAC) Humanitarian Assistance/Disaster Relief (HA/DR) missions, namely, a BLU-MED^®^ incremental modular equipment package along with a Rapid Deployment Medical Team (RDMT). The study was conducted to uncover a way for the U.S. Army to: (1) better medically support the greater U.S. military Pacific Command, (2) prepare the Army for Pacific HA/DR contingencies, and (3) imprint a swift presence and positive contribution to Pacific HA/DR operations.

**Methods:**

The findings were derived from an intensive quasi-Military Decision Making Planning (MDMP) process, specifically, the Oracle Delphi. This process was used to: (1) review a needs assessment on the profile of disasters in general and the Pacific in particular and (2) critically examine the viability and issues surrounding a Pacific HA/DR medical response of going in light and incrementally.

**Results:**

The Pacific area of operations contains 9 of 15 countries most at risk for disasters in the most disaster-prone region of the world. So, it is not a matter of whether a major, potentially large-scale lethal disaster will occur but rather when. Solid empirical research has shown that by every outcome measured Joint Forces (Army, Navy, Air Force, and Marines) medical HA/DR operations have been inordinately successful and cost-effective when they employed U.S. Army medical assets inland near disasters’ kinetic impact and combined sister services’ logistical support and expertise. In this regard, USARPAC has the potential to go in light and successfully fill a vital HA/DR medical response gap with the RDMT and a BLU-MED^®^. However, initially going in fast and light and expanding and contracting as the situation dictates comes with subsequent challenges as briefly described herein that must be addressed.

**Conclusions:**

The challenges to going in light are not insurmountable “show stoppers.” They can be identified and addressed through planning and preparation. Hopefully, the acquisition rapid response light components will equip commanders with more effective options with which to conduct Pacific HA/DR operations and be a focal point for effective joint operations.

## Background

According to a recent comprehensive United Nations (UN) 30-year retrospective study, the U.S. military’s Pacific Command (PACOM) region is one of the more, if not the most, disaster-prone areas in the world [1]. It encompasses 50% of the Earth’s surface and population and 10 of its largest militaries and is largely covered by water and sub-tropical coastal and mountainous remote interiors, with one of the Earth’s most massive shifting tectonic plates. It also hosts some of the most populous areas with rapid population growth, urbanization, and unrestrained expanding industry and increasingly crowded sea and land transit lines at high risk for large-scale manmade disasters [2, 3]. Not only are pure large-scale natural and manmade disasters likely, but substantial populations are concentrated astride areas that are predisposed to natural disasters with the potential for industrial disaster sequelae (e.g., earthquake/tsunami Fukushima nuclear meltdown). The Pacific region is extremely active and volatile seismically, meteorologically, volcanically, geologically, and industrially, especially with recent climate changes [4]. Disasters of epic proportions are not matters of if, but when, where, what, and how best to respond [5].

The UN’s Pacific Disaster report [1–6] provided an epidemiology of past disasters in the Pacific area that can be a fair predictor of future events and likely provides a road map on how best to respond.

First, natural disasters in PACOM will be one or more of four types: flood, storm, earthquake/tsunami, and landslide. They will likely occur westward and at some distance from the U.S. in Southwestern Asia, Southeastern Asia, or Northwestern Asia, with the most prone to disasters being Fiji, the Solomon Islands, Tonga and Vanuatu, Bangladesh, Brunei/Darussalam, Indonesia–Malaysia, Cambodia, and the Philippines. Poorer nations and peoples without the means to respond effectively will be disadvantageously affected. Second, loss of life and injury will be highest in earthquakes/tsunamis, storms, and floods. Third, survivable injuries will be trauma, though the major medical event will be asphyxiation. Thus, the medical surgical specialties needed will be trauma surgeons, orthopedic surgeons, and neurosurgeons, as well as nursing staff and support equipment. Fourth, reducing the number of deaths means getting medical “boots on the ground” nearest the epicenter of disasters’ maximum kinetic energy before the injured there die of survivable injuries. The epicenters will be inland from coastlines, airports, and seaports. Fifth, though all nations in the region have a disaster response plan, research suggests initially a U.S. joint military response will be the only viable and capable mechanism for delivering needed medical treatment quickly [7]—though this does not preclude other nation’s from sending HA/DR teams too. Sixth, transportation routes for patients and access routes for supplies will be compromised and all resources will have to be imported.

Thus, a short-notice (< 24 h) self-contained, pre-positioned westward trauma operating room structure with medical personnel that can also accommodate routine care is needed for immediate deployment. This structure should be integrated and interoperable seamlessly with civilian disaster response.

Research shows, particularly for Pacific area disasters, that emergency medical disaster relief: (1) must be able to quickly (no later than 72 h max) move and be fully operable proximate to epicenters where the greatest kinetic energy of a disaster occurred, but the quicker the better, (2) consist primarily of acute trauma and routine medical treatment, behavioral healthcare, some OB/PEDs capability, and, if needed, (3) be able to stabilize and transfer patients to higher levels of care [8–12]. Disaster relief must be <72 h post-request (but the sooner the better) for help or it likely becomes part of the problem (e.g., “in the way” or “a bottleneck”) [13, 14] An evaluation of the Pakistan earthquake revealed that not 1 of the 43 field hospitals arrived early enough, and they created a traffic jam when they all finally arrived at the same time [15]. Furthermore, everyone failed to bring the right kind of capabilities at the right time, including the U.S. military. Though the consensus on the Nepal disaster is still in progress, it would appear the more recent HA/DR responses repeated similar errors as in the past but to a lesser degree [16–21]. If anything, the Nepal experiences support the notion that the ability to quickly transport essential medical equipment and healthcare personnel to the nation’s interior were crucial to providing critical medical treatment [19, 20].

Conclusion: Emergency relief needs to be appropriate, rapid/immediate, and adaptable to the changing situation and augment existing hospital systems [15, 22–25]. Given a disaster has not made requesting help impossible, emergency disaster relief must be solicited by the nation in which the disaster occurred. But after that request has been received, disaster relief must come almost immediately; therefore, military contingencies must be prepared in advance for on-call service [26–29].

These efforts must be adaptable/scalable (i.e., modular) to conform to the unfolding situation and interoperable with local practitioners and existing higher level health/hospital infrastructures still operable or regaining operability in the area/region [27, 28]. Even though local medical infrastructure will be compromised, many local skilled medical practitioners will be available [25].

Sixty-five percent of disaster patient care will occur in remote/field areas not near air or sea ports [22]. Additionally, despite where disasters occur, inland or coastland, local health agencies, clinics, and hospitals will initially be compromised and overwhelmed but will be rebuilding. So, what is needed is an immediate surge of support and resources for the interim [13, 14].

Medical events will include lacerations, contusions, blunt force trauma, fractures, internal injuries, punctures, burns, asphyxiations, amputations, and obstetric complications [11] and births [10, 13, 14, 23, 24, 27]. Wound cleaning and dressing constitutes the majority of needs. Fifteen to twenty percent of patients will be emergent surgical and the majority will require routine care [8, 14] There is minimal need for preventive medicine; immediate post-disaster risk of infectious disease epidemics is over-exaggerated, though there is a need for prudent preventive medicine monitoring [29]. Nevertheless, long-term post-disaster preventive medicine is important to strengthening partnerships and alliances, providing security, and demonstrating the U.S. resolve to protect its interests in the region [30, 31]. It should be seamlessly woven into a gentle follow-on post-disaster transition.

Humanitarian Assistance/Disaster Relief (HA/DR) is a cornerstone of the U.S. military’s Pacific strategy which: (1) focuses on strengthening alliances and partnerships, (2) provides assurance of U.S. security commitment to the region, and (3) effectively communicates the U.S. resolve to protect its interests and ensure that the region remains stable and secure [6, 7, 30, 31]. The U.S. military’s global health engagements in the region support security and stability by building the capacity of military and civilian health systems to respond to disasters and health emergencies at the local, national, regional, and global levels [32]. Undoubtedly, disasters can provoke instability in any region. Therefore, U.S. military personnel stationed in PACOM have the ability to forward presence and crisis respond in terms of an array of contingencies, including humanitarian and medical assistance in response to a disaster [33]. Though the number of U.S. military responses to disasters in the region has been relatively small (<6%), each service must have the capability to effect a rapid medical response.

The U.S. Army through the U.S. Army Pacific (USARPAC) has the potential and is experienced in far-forward deployment in rugged and remote areas where the epicenter of a disaster is most likely to have occurred and its impact felt, during a time when an affected nation’s infrastructure is compromised [30]. The Army previously has relied on a “going-in heavy” strategy of methodically cobbling together large-scale teams and Medical Emergency Units (MEUs) or Combat Support Hospitals (CASHs), which are extremely bulky, hard-to-transport, self-contained units with operating and recovery rooms and patient wards [6, 15, 30, 32, 33]. Despite recent rapid advances in lighter, more modern, sturdier, and more mobile medical facilities and technologies, to date, the Army, in particular, USARPAC, has not had any in its inventory in terms of rapid HA/DR response. The classic doctrine for military operations for war is the use of methodical overwhelming force at the point of a center of gravity [34–36].

However, HA/DR operations doctrine stipulates that the proper approach is rapid response and initially a minimal footprint aimed at providing assistance and respite establishing a foundation for possible augmentation until local infrastructure can become operational again [22–24]. Past HA/DR medical operations (e.g., Pakistan earthquake) were large, and therefore cumbersome, ineffective, and costly, and became part of the problem instead of remedying it [13, 14] Thus, because the U.S. Army Pacific has been tasked with planning and preparing for HA/DR emergency medical operations, it has been exploring and developing its own particular alternative version of “going-in light” or “Going Light” that is fully integrate-able with its sister services in Joint Forces operations—generic but adaptable according to the specific situation.

Therefore, given the importance of this matter in terms of U.S. strategic interests and PACOM mission accomplishment, the intent of this article is to provide a report on study efforts to formulate a course of action, specifically, a viable, realistic, effective, relevant, PACOM/USARPAC, light-weight, highly mobile, and adaptably modular Rapid Emergency Medical Response capability. In so doing, this report will focus on equipment and personnel issues and then touch on ancillary aspects for further consideration. Hopefully, this report will serve as one crucial step forward toward achieving this as a Joint Forces/USARPAC capability.

## Methods

The Aim of the study efforts reported here in was to develop equipment and personnel relevant to the U.S. Army strategy of “going in light” regarding disaster relief in its Pacific Theater of Operations. To achieve this aim, the Oracle Delphi [37–39] process was the study method used to derive the information contained in this report. Although this process is not the preferred Military Decision Making Process (MDMP), when formulating prospective military plans and operations, the method includes desirable features of the MDMP in that group judgments and input are more valid than individual judgments [39]. This also compensates for shortfalls of other more isolated methods where precise prediction has yet to be established. For the study reported herein, a preliminary concept brief was initially compiled. This then was circulated among 10 different USARPAC Medical Plans and Operations subject matter experts, each with a different specialization area regarding Army medical operations in PACOM. Each expert anonymously reviewed the original product and provided written opinions. This was repeated until a general consensus was reached. Anonymity was preserved to prevent authorities, personalities, and reputations from dominating or biasing the process, to permit unfettered opinions and open critique, and to facilitate admission of errors and reformulation.

## Results/discussion

Disaster “ground rules” govern the requirements for any USARPAC rapid deployment medical response for an HA/DR situation. These rules dictate that the unit be responsive, portable, and modular for “plug-in-and-play.” Specifically, it should have the built-in capacity to balance personnel and equipment on the spot in response to situational contingencies. Including the time needed to arrive on scene and become fully operational, it should be off-the-shelf, that is, rapidly delivered, placed, and operating (boots on the ground <72 h from alert and <24 h on order). Otherwise, it becomes part of the problem. It needs to be scalable, either up or down—up for long-term and down for short-term missions—and able to provide at least a Level 1 and 2 care facility. Also, it must add value to other sister services as part of Joint Forces capabilities.

### Commercial off-the-shelf package and an HA/DR special team

**Fig. 1:**
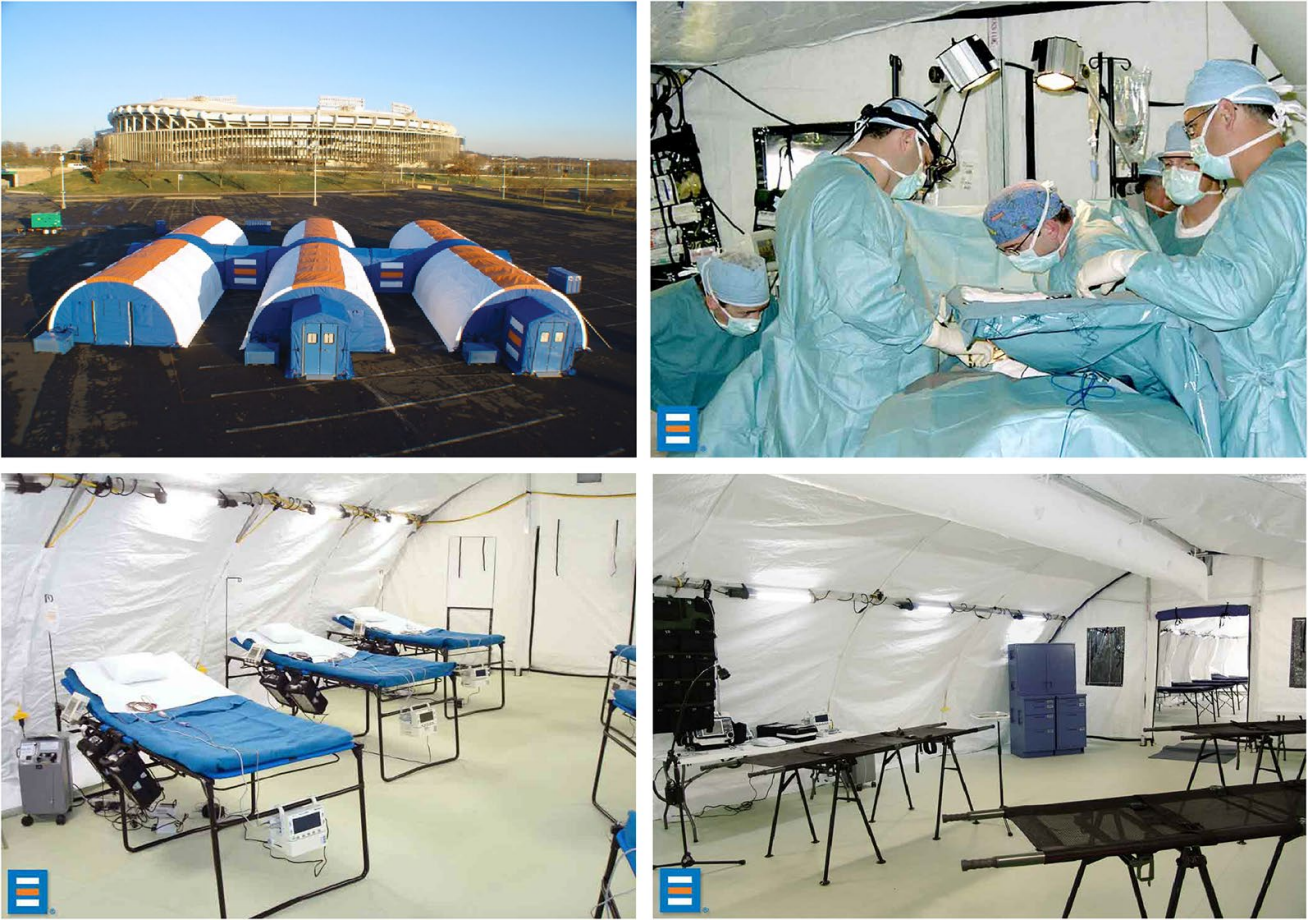
BLU-MED^®^ response systems mobile medical facility

**Fig. 2:**
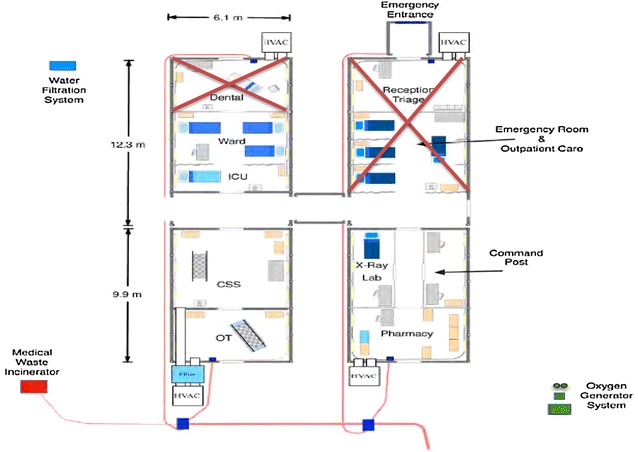
Phase 1 4-bed facility

**Fig. 3:**
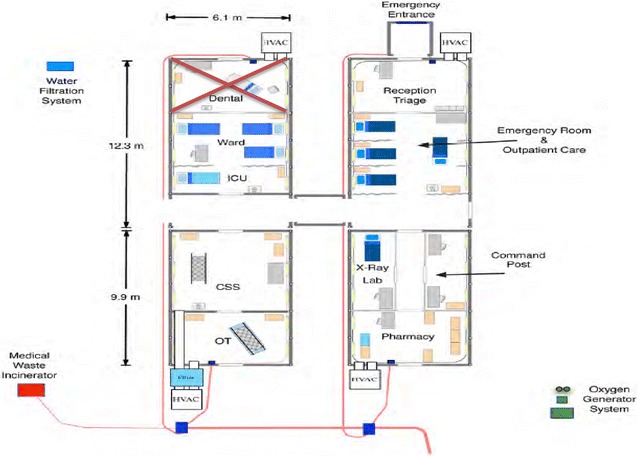
Phase 2 10-bed facility [Dental would be excluded as this is typically not part of Disaster Relief missions. Omitting Dental also drops the price of the package substantially such that the price of a 25 bed facility is the same as a 10 bed facility ($1.6 million)]

The aforementioned limitations, complexities, and de-conflictions lead the Study Group to support a radical (i.e., hybrid/“outside the box”) alternative course of action and then iteratively consider and explore/develop its potential viability. Specifically, the Study Group floated the idea of a commercial off-the-shelf compact equipment package stored and maintained in Hawaii and staffed from Medical Command (MEDCOM) Regional Health Command Pacific, preferentially, Tripler U.S. Army Medical Center. Just such a forward pre-positioned, pre-package (i.e., “hospital in a box”—or rather two boxes) can be purchased from BLU-MED^®^ Response Systems (see Figs. 1, 2, 3), which provides 4- to 25-bed facilities.

The facilities BLU-MED^®^ offers are fully equipped mobile, portable, flexible/modular medical treatment facilities for United Nations and other peacekeeping medical treatment operations.

BLU-MED^®^ is also the only provider of such systems. Its systems are 100% portable, compact, and permit multi-transportation options. A small team of Soldiers can easily and rapidly assemble them (“snap together”) ‘in a couple of hours’ with minimal training provided by BLU-MED^®^. They have been used by the United Nations and Centers for Disease Control and Prevention in situations similar to HA/DR (e.g., Ebola in West Africa) with great reviews [40]. They allow for flexible configuration and are scalable in response to either expanding or diminishing situations. They are far lighter and more durable than the classic Army MEU (“conex”) or even CASH. The BLU-MED^®^ modules that were considered optimal for a U.S. Army immediate response capability (<24 h “boots on the ground” fully operational) were: 4-bed facility, Role II+ (−) Care. 1 ICU. 3 ICWs, 1 OR Beds, and/or depending on the extent of the situation, 10-bed facility, Role II+ (−) Care, 2 ICU, 8 ICW, 1 OR Beds, PLX, CP (see Figs. 2, 3).

An extension of a 10-bed facility can be added for temporary housing for troops. Thus, a 25-bed facility would cost approximately the same as the 10-bed facility. Also, the modularity of the systems permits separating the medical treatment sets from the tent units and leaving the tents behind in case there is an available existing and non-compromised structure (e.g., school, church, government building) near a disaster’s epicenter in which to conduct medical treatment. But the modular tents provide an option to avoiding becoming victim to structures prone to immediate post-disaster hazards and “ripple effects, for example, earthquake aftershocks. Whatever the case, this feature upholds two paramount tenets of immediate disaster response, which are to use available infrastructure first and going in light but effective [41–43].

This system and its proposed personnel staffing conform to the disaster management principal of minimalism, specifically, minimizing the intrusiveness and imposition (i.e., “footprint”) of disaster relief and delivering the right amounts at the right time and increments/decrements in response to the unfolding situation. The staffing recommendation is equally light depending on the size of the BLU-MED^®^ system (see Tables 1 and 2). “Going in light” also means substantial “dual hatted” roles (i.e., “double tapping”), for example, personnel will have to cover down on both pre- and post-op, thus they must have trauma experience. And since it is “going in light” the expectation is that it is not designed for long-term/indefinite continuous operations without substantial augmentation and relief.

**Table 1:** Staffing requirements 4 bed facility

1 X 61 J General Surgeon w/Trauma experience
1 X 61 M Orthopedic Surgeon
1 X 66F Nurse Anesthetist
1 X 68Q Pharmacy Specialist
1 X 66S Critical Care Nurse w/behavioral health experience
1 X 66H Medical Surgical Nurse/Trauma experience
1 X 68D OR Tech
1 X 66P Family Nurse Practitioner w/behavioral health experience
1 X 68C Practical Nursing Specialist
2 X 68 W Health Care Specialists one dual-hatted for supply
1 X 68P Radiology Specialist
1 X 68 K Laboratory Specialist
13 Total
Possibly expanded to 15 with other specialties per mission requirements

If there is a need for Preventive Medicine personnel, they will be TACON and OPCON to the Teams, but ADCON to a higher Command Level. Research shows that Preventive Med is needed more Pre-and-Post Disaster

The Numbers/Letters are U.S. Army (Medical) Military Occupational Specialty (MOS) designations denoted by their respective corresponding job title

**Table 2:** Staffing requirements 10 bed facility*

1 X 61 J General Surgeon w/Trauma experience
1 X 61 M Orthopedic Surgeon
1 X 70H Planning, Admin, OIC, and dual-hatted PAO
1 X 66F Nurse Anesthetist
1 X 68Q Pharmacy Specialist
2 X 66S Critical Care Nurse w/behavioral health experience
1 X 66H Medical Surgical Nurse/trauma experience
2 X 68D OR Tech
1 X 66P Family Nurse Practitioner w/behavioral health experience
2 X 68C Practical Nursing Specialist
4 X 68 W Health Care Specialists one dual hatted for supply
1 X 68P Radiology Specialist
1 X 68 K Laboratory Specialist
19 Total
Possibly expanded to 25 other specialties per mission requirements

The primary staffing source would be Tripler Army Medical Center—pre-identified and tasked by name with a special Medical Occupational Specialty (MOS) training identifier as HA/DR qualified. Some will be “dual-hatted” in terms of medical specialization: Phase 1 Facility 13–15 Personnel (PAX) and Phase 2 Facility 19–25 PAX.

Manning and the manning process would draw on the Special Medical Response Command-Pubic Health Personnel Capabilities Model [43]. That is, it would be capable of deploying all year round to respond to and assist in humanitarian disasters, both regional and domestic. An initial liaison/coordinating element (a Rapid Response Officer) would be established within 12 h of being alerted. Tailored to situation requirements, particular specialists would be deployed within a minimum of 24 h and a maximum of 72 h of being alerted. Personnel would be capable of self-sustaining for up to 72 h with organic equipment, supplies, and meals without any re-supply from other units or organizations. Sustainment support would be required for austere-environment deployments after the 72-h initial window.

The Rapid Disaster Medical Response Team (RDMT) and BLU-MED^®^ would be co-located at or near Tripler Army Medical Center in Hawaii for forward positioning, easy mobilization, and proximity to sister services for quick transport. The Study Group conceived of the RDMT with BLU-MED^®^ as a “quick grab-it-and-go first aid” or “band-aide stop-gap” approach to HA/DR disaster relief until: (1) an actual on-the-scene assessment of the situation can be conducted and a decision rendered regarding mission expansion or (2) the local medical treatment infrastructure comes back on line. Nevertheless, BLU-MED^®^ can also provide equipment which local health providers can use.

However, this “Going in Light” option is not a panacea without dependencies and limitations, such as transportation, material handling equipment, sustainment, maintenance (general/biomedical), pharmaceuticals, and assemblage training and ancillary considerations.

### Transportation

This system and its personnel will need a ride to and from wherever it is they are going. If medical evacuation and transport are involved, then there needs to be ground transportation and a nearby airfield. Also, in and around transportation will be needed.

### Material handling equipment

As light and mobile as the equipment is, it still is heavy enough to require heavy equipment to move it. Naturally, equipment must be pre-arranged (i.e., pre-packed) for easy deployment to the area of operations.

### Sustainment

These systems and eventually personnel will need logistical support and to be tied into a logistical re-supply line (Class I, III, VIII). Patients will need sustainment support as well. Note that the generators are lightweight but this also means more of them are needed and each is a relatively substantial fuel consumer (3–4 gallons per hour per generator). So, substantial fuel re-supply and storage must be available. However, the fact these power systems are self-contained means they are not dependent on local power sources, which might be compromised anyway and just as likely not suitable for U.S. equipment.

### Maintenance (general/biomedical)

Whether stored in a warehouse in Hawaii or deployed to an actual disaster epicenter, eventually, the systems must be maintained and/or reconstituted. The question always is who will be responsible for maintenance and funding maintenance.

### Pharmaceuticals

No off-the-shelf equipment systems come with either pharmaceuticals or respiratory gases. These are added expenses. Note that the disaster pharmaceutical surgical and routine medical treatment sets are also an added expense. BLU-MED^®^ does not provide those as part of its BLU-MED^®^ packages. However, they are reasonably inexpensive and easily obtained through the Tripler U.S. Army Medical Supply system (30 days’ supply of routine treatment <$50,000 and surgical treatment <$5000).^1^ However, the surgical drug sets contain substantial amounts of Drug Enforcement Administration (DEA)-scheduled medications that must be monitored and controlled. Both sets can be packaged and available for pickup from Tripler within 24 h of alert/request.

### Assemblage training

Also, ongoing assemblage training is necessary. As part of the acquisition package, BLU-MED^®^ provides only a one-time assembly training session. After that, it would be the responsibility of the HA/DR Rapid Response Team to pass down the BLU-MED^®^ assembly training to new members by conducting regular assemblage training. Alternatively, this training would have to be contracted from BLU-MED^®^ at an additional expense. Training events where the BLU-MED^®^ is taken off the shelf and put into simulated action also could be opportunities to practice and coordinate with possible host nations and regional militaries (e.g., National Guard State Partnerships) [44] and HA/DR civilian officials. Furthermore, this would also dispel a possible criticism, that is, if the U.S. Military only responds to 6% of the disasters in the region, its acquisition hardly justifies the investment however small.

### Other ancillary considerations

Also, other ancillary considerations must be considered in terms of the viability of the BLU-MED^®^/RDMT option but these would be incumbent on any HA/DR emergency medical operation, whether BLU-MED^®^ is employed or not. Security force protection will be needed and can be provided by the enlisted Soldier Emergency Medical Technicians (EMTs-68Ws). However, this should be tasked to security specialists (e.g., U.S. Marines or Air Force Security Police). Just because the U.S. Army is there to help does not mean that its presence may not afford a target of opportunity. A source for water will be needed though the BLU-MED^®^ system does have a water purification system. There also needs to be provision for sanitation and waste removal. BLU-MED^®^ has incineration capabilities for bio-waste. The BLU-MED^®^ systems, though compact, when fully deployed along with transportation avenues still require substantial amounts of real estate.

**Fig. 4:**
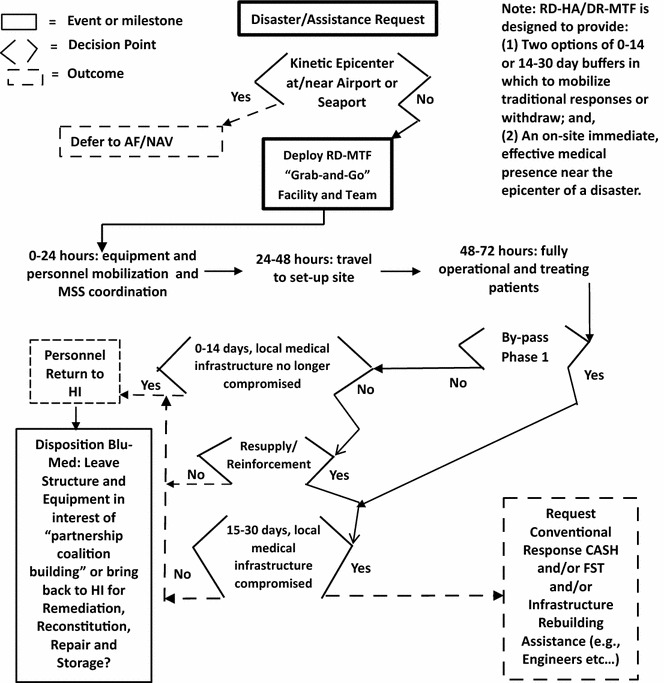
Tree decision model for PACOM RDMT for HA/DR

Therefore, depending on the particular disaster situation, cut-points (“Rubicon-s”; see Fig. 4) must be pre-established for BLU-MED^®^ size (Phase 1 or 2), BLU-MED^®^ expansion (Phase 1–2), mission expansion (follow-on with a complete Combat Support Hospital), or mission withdrawal.

Additionally, a communication (signal) system must be provided for voice and data. One suggestion was the use of contracted light-weight satellite cell phones as the missions were unclassified. Data also include the particular system to document medical treatment and track in- and out-patients and patient transport. Adequate high-speed electronic communications also means telemedicine could be employed as a force-multiplier.

Finally, the disposition of the BLU-MED^®^ facility post-mission must be considered. Specifically, (1) will it be rolled up, boxed up, and shipped back to Hawaii or (2) will it be left in place in the interest of local service/training and forging military-civil Pacific partnerships, or will some other disposal be used to avoid the inconvenience and expense of return shipment? Donation would improve the medical systems near U.S. and multinational forces, thus fostering self-sufficiency and contributing to the earlier achievement of the U.S. PACOM military mission [15, 22]. However, some sensitive components such as controlled medications and electronics would have to be packed and shipped back regardless.

## Conclusion

This article reported preliminary yet intensive study efforts aimed at formulating an “out-of-the-box” course of action, specifically a viable, realistic, effective, relevant, PACOM/USARPAC, light-weight, highly mobile, and adaptably modular Rapid Emergency Medical Response capability. In so doing, this report focused primarily on equipment and personnel issues and then touched briefly on ancillary aspects for further and more definitive consideration. PACOM’s area of operations contains 9 of 15 countries most at risk for disasters in the most disaster-prone region of the world [31]. USARPAC is the Department of Defense’s subject matter expert in PACOM on inland HA/DR operations proximate to the kinetic epicenter of most disasters. However, USARPAC does not even have a rapidly deployable Level 1 and 2 medical capability available for HA/DR relief. In conjunction with sister services for supply and forward projection and delivery systems (e.g., Blue Pathways) in Joint Forces operations, the Rapid Disaster Medical Response Team with a BLU-MED^®^ commercial off-the-shelf package provides a rapid, on-the-spot, flexible, and responsive solution to HA/DR situational contingencies. Also, solid empirical research shows that this Joint Forces scenario has been the most successful.

Though this system is not without challenges, some unique to the components and system described and some generic to “Going in Light” disaster relief, they are not insurmountable “show-stoppers.” They can easily be identified through disaster preparation exercises, anticipated and addressed far in advance, and accounted for in operations’ planning. One other consideration in terms of the way forward is in the future encouraging host-nations prone to disaster to purchase their own BLU-MED^®^ and assembling an RDMT in the interest of accessibility, immediacy, and inter-operability and collaboration. Nevertheless, the BLU-MED^®^/RDMT and “going in light” is not a single solution/approach but one that incorporates development, evolution, augmentation, and incremental responsiveness given the unfolding situation.

Hopefully, the acquisition of the BLU-MED^®^ and formation of the RDMT will equip commanders with more and more effective options with which to conduct Pacific HA/DR operations. Also, the expectation is that this new USARPAC capability will be the impetus for more Joint Forces operations and will serve as a centerpiece for facilitating joint training with multi-national PACOM partner nations in the interest of regional stability. Future program evaluation should investigate its cost-effectiveness and deploy-ability, public relations potential, and creative ways in which ancillary challenges related to it can be addressed.

## Abbreviations

$: American dollars
AF: Air Force
BLU-MED: medical response package/emergency shelters
CASH/CSH: combat army support hospitals
Class I: rations—subsistence (food and drinking water)
Class III: petroleum, oils, lubricants
Class VIII: medical material (equipment and consumables)
Conex: container express/large metal self-contained shipping container
CP: command post
CSS: combat service support/support services also pre-op prep area
DEA: Drug Enforcement Agency
DoD: (United States) Department of Defense
EMT: emergency medical technicians
ER: emergency room
FST: Forward Surgical Team
H: hour
HADR or HA/DR: Humanitarian Assistance Disaster Response
HVAC: heating ventilation air-conditioning
HI: Hawaii
ICU: intensive care unit
ICW: intensive care ward
MDMP: Military Decision Making Process
MEDCOM: (U.S. DoD) Medical Command
MEU: medical emergency units
MOS: (U.S. Army) Military Occupational Specialty Codes
MTF: medical treatment facility
MSS: medical service supply
NAV: Navy
OR: operating room
OT: outpatient treatment
PACOM: (United States Department of Defense) Pacific Command
PAX: personnel
PLX: pharmacy/lab/X-ray
RD: paid deployment
RDMT: Rapid Deployment Medical Team
U.S.: United States
UN: United Nations
USARPAC: United States Army Pacific Area Command

## Footnotes

1 Note: Dollar amounts are as of 2016.

## References

[1] UN ESCAP: Overview of natural disasters and their impacts in Asia and the Pacific, 1970–2014, ESACP Technical paper information paper and communications technology and disaster risk reduction division, 7 March 2015. p. 1–8, 22–7.

[2] UN ESCAP: Overview of natural disasters and their impacts in Asia and the Pacific, 1970–2014, ESCAP Technical paper information paper and communications technology and disaster risk reduction division, 7 March 2015. p. 24–5.

[3] Asian Development Bank (2011). Financial integration and capital flow volatility in emerging asia: issues and policies.

[4] Asian Development Bank (2011). Adapting to climate change: strengthening the climate resilience of the water sector infrastructure in Khulna, Bangladesh.

[5] Simpson A, Cummins P, Dhu T, Griffin J (2008). Assessing natural disaster risk in the Asia-Pacific region. AusGeo News..

[6] Andrews RJ, Quintana LM (2015). Unpredictable, unpreventable and impersonal medicine: global disaster response in the 21st century. EPMA J.

[7] Smart T. Designed for war, honed in disaster: ADF AME in the Asia-Pacific region. Australia defense force air medical evacuation presentation at the aerospace medical association annual meeting, San Diego, 2014.

[8] Awais S, Saeed A (2013). Study of severity of musculoskeletal injuries and triage during the 2005 Pakistan earthquake. Int Orthop.

[9] Barboza P, Coulombier D, Defilippi L (2006). Outbreak of tetanus cases following the tsunami in Aceh Province, Indonesia. Glob Public Health.

[10] Eyre A (2008). Meeting the needs of people in emergencies: a review of UK experiences and capability. Emerg Health Threats J.

[11] van Walsum AD, Rodel SG, Klaase JM (2001). Local and regional in-hospital trauma care following fireworks depot explosion in Enschede. Nederlande Tijdschr Geneeskd.

[12] Haeri S, Marcozzi D (2015). Emergency preparedness in obstetrics. Obstet Gynecol.

[13] Noe RS, Schnall AH, Wolkin AF (2013). Disaster-related injuries and illnesses treated by American Red Cross disaster health services during Hurricanes Gustav and Ike. South Med J.

[14] McIntyre T, Hughes CD, Pauyo T (2011). Emergency surgical care delivery in post-earthquake Haiti: partners in Health and Zanmi Lasante experience. World J Surg.

[15] von Schreeb J, Riddez L, Samnegard H (2008). Foreign field hospitals in the recent sudden-onset disasters in Iran, Haiti, Indonesia, and Pakistan. Prehosp Disaster Med.

[16] Asokan GV, Vanitha A (2016). Disaster response under one health in the aftermath of Nepal earthquake, 2015. J Epidemiol Glob Health.

[17] Yamamoto T (2015). Participation in relief activities in the aftermath of the Great Nepal earthquake and disaster reconstruction assistance. Japan Med Assoc J.

[18] Nielsen MJ, Ferguson S, Joshi AK, Rimal S (2016). Post-earthquake recovery in Nepal. Lancet Glob Health.

[19] Merin O, Yitzhak A, Bader T (2015). Medicine in a disaster area: lessons from the 2015 earthquake in Nepal. JAMA Intern Med.

[20] Ho ML, Lim JZ, Tan MZ, Kok WL (2016). Humanitarian Assistance and Disaster Relief mission by a tripartite medical team led by the Singapore Armed Forces after the 2015 Nepal earthquake. Singap Med J.

[21] Basnyat B (2016). Post-earthquake Nepal: acute-on-chronic problems. Natl Med J India.

[22] FEMA. The Federal Emergency Management Agency; 2010. p. 30–5.

[23] FEMA. FEMA Management and Support Keystones; 2011. p. 19–43.

[24] NIMS. National Incident Management System. Homeland Security; 2008. p. 5–7, 21.

[25] de Ville de Groyet C (2007). Health lessons learned from the recent earthquakes and Tsunami in Asia. Prehosp Disaster Med.

[26] Campos-Outcalt D (2006). Disaster medical response: maximizing your effectiveness. J Fam Pract.

[27] Pesik N, Keim M (2002). Logistical considerations for emergency response resources. Pac Health Dialogue.

[28] Yamada S, Gunatilake RP, Roytman TM (2006). The Sri Lanka tsunami experience. Disaster Manag Response.

[29] Klein KR, Pepe PE, Burkle FM (2008). Evolving need for alternative triage management in public health emergencies: a Hurricane Katrina case study. Disaster Med Public Health Preparedness.

[30] Moroney JD, Pezard S, Miller, LE, Engstrom J, et al. Lessons from Department of Defense disaster relief efforts in the Asia-Pacific region. RAND Corporation, Prepared on behalf of the Department of Defense, 2013. p. 24.

[31] Chinn CG Adm. Humanitarian Assistance and Disaster Relief in the Indo-Asian-Pacific. EMC chair conference paper. https://www.usnwc.edu/Academics/Faculty/Derek-Reveron/Workshops/Maritime-Security,-Seapower,—Trade-(1)/papers/chinn.aspx.

[32] Moroney JD, Pezard S, Miller, LE, Engstrom J, et al. Lessons from Department of Defense disaster relief efforts in the Asia-Pacific region. RAND Corporation, Prepared on behalf of the Department of Defense. 2013. p. 41–47.

[33] Moroney JD, Pezard S, Miller, LE, Engstrom J, et al. Lessons from Department of Defense disaster relief efforts in the Asia-Pacific region. RAND Corporation, Prepared on behalf of the Department of Defense. 2013. p. 39–41.

[34] Corum J (1992). The roots of Blitzkrieg: Hans von Seecht and German military reform.

[35] Johnston P (2000). Doctrine is not enough: the effect of doctrine on behavior of armies. Parameters..

[36] Clausewitz CV. Chapter 1: What is war? In: Howard M, Paret P, editors and trans. On war, vol. 16. Princeton: Oxford University Press. 1976. p. 16, 23–25.

[37] Rowe G, Wright G (1999). The Delphi technique as a forecasting tool: issues and analysis. Int J Forecast.

[38] Rowe G, Wright G, Armstrong JS (2001). Expert opinion in forecasting: role of Delphi technique. Principles of forecasting: a handbook of researchers and practitioners.

[39] Dalkey N, Helmer O (1963). An experimental application of the Delphi method to the use of experts. Manag Sci.

[40] http://blu-med.tumblr.com/post/108274463128/blu-meds-mobile-field-hospitals-and-ebola.

[41] Wade N. BSS5: battle staff SMARTbook, 5th rev. ed. (leading, planning & conducting military operations). The Lightning Press, SMARTbooks; 2015.

[42] Lane DA (2006). Medical support to Sri Lanka in the wake of tsunamis: planning considerations and lessons learned. Mil Med.

[43] https://phc.amedd.army.mil/organization/phcrpac/Pages/SpecialMedicalResponseCapability-PublicHealth.aspx.

[44] http://www.nationalguard.mil/Leadership/Joint-Staff/J-5/International-Affairs-Division/State-Partnership-Program/.

